# 

**Published:** 1853-02

**Authors:** 


### CONTENTS

### OF NO II.

### ORIGINAL COMMUNICATIONS.

### ESSAYS AND CASES.

ART. I.—Discourse on the Life, Character, and Services of Dr. Daniel Drake.—Delivered, by request, before the Faculty and Medical Students of the University of Louisville, January 27, 1853. By S. D. GROSS, M. D., Professor of Surgery. - - - - - - - - - 95

ART. II.—Contributions to the History, Diagnosis, and Management of Croup. BY JOHN WARE, M. D., Professor of the Theory and Practice of Medicine in Harvard University. - - - - - - - - - - - 167

Treatment of Croup. Read before the Boston Society for Medical Improvement, by JOHN WARE, M. D., Nov. 11, 1844. - - - - - - - - - 172

Further Remarks on the Treatment of Croup. Read before the Boston Society for Medical Improvement, Feb. 20, 1845. 178

Additional Remarks on the Treatment of Croup. Read before the Suffolk District Medical Society, March, 1850. 183

### SELECTIONS FROM FOREIGN AND HOME JOURNALS

Abortion without suspicion of Pregnancy. - - - - - 186

### ORIGINAL INTELLIGENCE.

University of Louisville. - - - - - - - - - 187

Discourse on Dr. Drake. - - - - - - - - - 188

New Books and Communications. - - - - - - - 188

